# LncRNA *TubAR* complexes with TUBB4A and TUBA1A to promote microtubule assembly and maintain myelination

**DOI:** 10.1038/s41421-024-00667-y

**Published:** 2024-05-21

**Authors:** Xiaolin Liang, Meng Gong, Zhikai Wang, Jie Wang, Weiwei Guo, Aoling Cai, Zhenye Yang, Xing Liu, Fuqiang Xu, Wei Xiong, Chuanhai Fu, Xiangting Wang

**Affiliations:** 1 Department of Geriatrics, Gerontology Institute of Anhui Province, Centre for Leading Medicine and Advanced Technologies of IHM, The First Affiliated Hospital, Division of Life Sciences and Medicine, University of Science and Technology of China, Hefei, Anhui China; 2 MOE Key Laboratory for Membraneless Organelles and Cellular Dynamics, Hefei National Science Center for Physical Sciences at Microscale & University of Science and Technology of China, School of Life Sciences/Division of Biomedical Sciences, Hefei, Anhui China; 3 Songjiang Hospital and Songjiang Research Institute, Shanghai Key Laboratory of Emotions and Affective Disorders, Shanghai Jiao Tong University School of Medicine, Shanghai, China; 4 Institute of Neuroscience and Brain Diseases, Xiangyang Central Hospital, Affiliated Hospital of Hubei University of Arts and Science, Xiangyang, Hubei China; 5 State Key Laboratory of Magnetic Resonance and Atomic and Molecular Physics, Key Laboratory of Magnetic Resonance in Biological Systems, Wuhan Center for Magnetic Resonance, Wuhan Institute of Physics and Mathematics, Innovation Academy for Precision Measurement Science and Technology, Chinese Academy of Sciences, Wuhan, Hubei China; 6 Center for Excellence in Brain Science and Intelligence Technology, Chinese Academy of Sciences, Shanghai, China

**Keywords:** Microtubules, Long non-coding RNAs

## Abstract

A long-standing hypothesis proposes that certain RNA(s) must exhibit structural roles in microtubule assembly. Here, we identify a long noncoding RNA (*TubAR*) that is highly expressed in cerebellum and forms RNA–protein complex with TUBB4A and TUBA1A, two tubulins clinically linked to cerebellar and myelination defects. *TubAR* knockdown in mouse cerebellum causes loss of oligodendrocytes and Purkinje cells, demyelination, and decreased locomotor activity. Biochemically, we establish the roles of *TubAR* in promoting TUBB4A–TUBA1A heterodimer formation and microtubule assembly. Intriguingly, different from the hypomyelination-causing mutations, the non-hypomyelination-causing mutation TUBB4A-R2G confers gain-of-function for an RNA-independent interaction with TUBA1A. Experimental use of R2G/A mutations restores TUBB4A–TUBA1A heterodimer formation, and rescues the neuronal cell death phenotype caused by *TubAR* knockdown. Together, we uncover *TubAR* as the long-elusive structural RNA for microtubule assembly and demonstrate how *TubAR* mediates microtubule assembly specifically from αβ-tubulin heterodimers, which is crucial for maintenance of cerebellar myelination and activity.

## Introduction

Microtubules are principal structural components of cells and play crucial roles in cell morphology maintenance, cargo trafficking or intracellular transport, cell division, and cell viability^1^. In 1977 and 1980, two pioneering studies showed that microtubule nucleation and structure of purified centrosomes were severely impaired by RNase treatment, suggesting that certain RNA(s) must play critical roles in supporting structural integrity of microtubule-based cytoskeleton^2,3^. Later on, another two studies also reported a dramatic disruption of mitotic spindle assembly by RNase treatment^4,5^. These findings suggest that RNA may function directly as a structural organizer in microtubule-based apparatus formation. However, such structural RNAs are yet-to-be discovered^6,7^.

Long noncoding RNAs (lncRNAs) are a group of functional macromolecules that participate in a wide range of physiological and pathophysiological events^8–21^. In the central nervous system (CNS), lncRNAs have been implicated in brain development, neuronal and glial function, and synaptic plasticity^13,17,20,21^.

The basic linear protofilaments of microtubule are structures built from α- and β-tubulin isotypes. Proteins of these two classes of tubulins assemble head-to-tail, and the formed filaments later self-organize into hollow tube structure of microtubules. Interestingly, the assembly kinetics of microtubule may vary due to the involvement of different tubulin isotypes^22,23^. In the CNS, microtubule-mediated processes are extensively involved in the functional regulation of both neurons and glia cells^24–32^. Of note, tubulin mutations can cause a group of brain malformations known as tubulinopathies^33,34^.

Among the tubulin superfamily, tubulin alpha-1a chain (TUBA1A) and tubulin beta-4a chain (TUBB4A) have been linked to cerebellum-related disorders^34–45^. A study established a correlation between certain TUBB4A mutations, including p.Asp249Asn (D249N), p.Val255Ile (V255I), p.Arg282Pro (R282P), and p.Asn414Lys (N414K) with its dominant toxic gain-of-functions on microtubule dynamics^46^. However, the p.Arg2Gly (R2G) of TUBB4A displayed normal tubulin quantity and polymerization, as well as normal oligodendrocyte morphology^46^. For the purposes of our discussion, we therefore classify the R2G mutation as the non-hypomyelination-causing TUBB4A mutation for comparison with those hypomyelination-causing TUBB4A mutations (D249N, V255I, R282P, and N414K). The underlying mechanisms for the different effects on the tubulin polymerization of these two groups of TUBB4A mutations are currently enigmatic.

Here, we identified a lncRNA-*TubAR* (tubulin-associated lncRNA) that is highly expressed in the cerebellum and interacts with the cerebellum/hypomyelination disease-related isotypes TUBA1A and TUBB4A. RNA immunoprecipitation (RIP) and protein co-immunoprecipitation (co-IP) analyses revealed an RNA–protein complex containing *TubAR*, TUBB4A, and TUBA1A. *TubAR* is required for TUBB4A–TUBA1A interaction and microtubule assembly. Knockdown of *TubAR* induces cell death of both neurons and oligodendrocytes. The hypomyelination-causing TUBB4A variants failed to interact with *TubAR* and TUBA1A. Conversely, the non-hypomyelination-causing variant R2G/A of TUBB4A possesses constitutively binding activity to TUBA1A. Taking advantage of this unique feature of mutations at TUBB4A’s p.Arg2 residue, we discovered that *TubAR* knockdown-triggered cell death is a consequence of TUBB4A–TUBA1A heterodimer disruption. Moreover, specific knockdown of *TubAR* in the murine cerebellum leads to demyelination, loss of oligodendrocytes and Purkinje cells, and decreased locomotor activity. Together, our work uncovers *TubAR* as the long-sought ‘structural RNA’ that promotes tubulin heterodimer formation and microtubule assembly, and reveals a previously unknown action of lncRNA in regulating cytoskeletal apparatus to ultimately impact brain functions.

## Results

### Identification of *TubAR* specifically expressed in the cerebellum

**Fig. 1:**
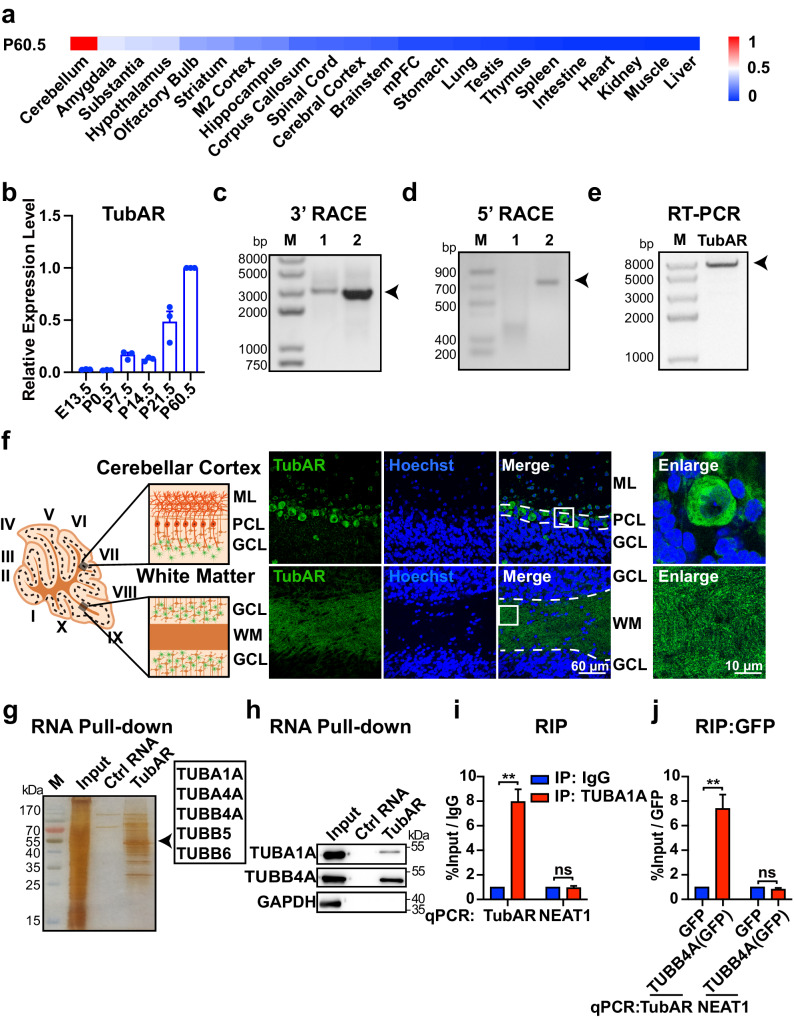
Identification of *TubAR* in cerebellum. **a** Heatmap of *TubAR* (*AK035765*) expression levels in the indicated adult murine tissues (*n* = 3), detected by qPCR. All data were normalized to *Gapdh*. **b** *TubAR* expression levels in different developmental stages (P0.5, P7.5, P14.5, and P60.5) in the cerebellum or in the metencephalon (E13.5) (*n* = 3), detected by qPCR. All data were normalized to *Gapdh*. **c**–**e** Electrophoretic gel image of 3′ rapid amplification of cDNA ends (RACE) (**c**), 5′ RACE (**d**) and full-length reverse transcription (RT)-PCR (**e**) for *TubAR* (murine cerebellum). **f** Left: Schematic illustration of the microscopic analytic region; right: FISH of *TubAR* (*AK035765*) in the cerebellar cortex or in the cerebellar white matter. Slice thickness, 6 µm. **g** Silver staining of electrophoretic gel image following RNA pull-down for *TubAR-*associated proteins (murine cerebellum extracts). Precipitated tubulins are indicated by the arrow head. **h** RNA pull-down assay to validate interactions between *TubAR* and TUBA1A and between *TubAR* and TUBB4A, using murine cerebellum extracts. GAPDH was used as a negative control. **i**, **j** RIP of endogenous TUBA1A (**i**) and overexpressed GFP-TUBB4A (**j**) in cultured Neuro-2a cell extracts (*n* = 3), showing the interactions of *TubAR* with TUBA1A (**i**) and GFP-TUBB4A (**j**). IgG and GFP, negative antibody controls. *NEAT1*, a negative lncRNA control for qPCR. Data are represented as means ± SEM. Statistical significance was determined using unpaired *t*-test with Welch’s correction; ns, *P* > 0.05; **P < 0.01. *P* postnatal days, E embryonic days, M marker, ML molecular layer, PCL Purkinje cell layer, GCL granule cell layer, WM white matter.

This study was initiated in an attempt to identify lncRNAs that regulate the cerebellar motor functions. To this end, we examined a set of our previously identified lncRNAs that are highly expressed in the adult mouse cerebellum^47^. Among these transcripts, we noted a lncRNA transcript, annotated as *AK035765*, a 7.3-kb transcript located on chromosome 5, and exhibit increased expression level during cerebellum development (Fig. 1a–e; Supplementary Fig. S1a, b). To characterize which cell type(s) express *TubAR* in the cerebellum, we carried out RNA fluorescence in situ hybridization (FISH) on mouse cerebellar sections. We observed strong *TubAR* signals in the cytoplasm of Purkinje cells and in white matter of the cerebellar vermis where nerve fiber bundles are gathered and myelinated by oligodendrocytes (Fig. 1f).

Next, we conducted RNA pull-down from murine cerebellar extracts followed by mass spectrometric analyses, which detected multiple tubulin isotypes as putative *AK035765*-interacting proteins, including TUBA1A, TUBA4A, TUBB4A, TUBB5, and TUBB6 (Fig. 1g; Supplementary Table S1). Given its capacity to interact with tubulins, we named *AK035765* as tubulin-associated lncRNA (*TubAR*). TUBB4A and TUBA1A have been implicated in cerebellum/hypomyelination diseases^34–36^, we, therefore, focused on these two isotypes and validated their interactions with *TubAR* through RNA pull-down and subsequent western blotting assay using cerebellar extracts (Fig. 1h). Consistent with its cytoplasmic expression in Purkinje cells and oligodendrocytes (Fig. 1f), we detected strong cytoplasmic *TubAR* signals in proliferating and differentiated Neuro-2a cells (cultured mouse neuroblastoma cell line) as well as in Oli-neu cells (cultured mouse oligodendroglial precursor cell line) by FISH analyses (Supplementary Fig. S2a–c). Next, we performed RIP assays with Neuro-2a cells expressing GFP-TUBB4A fusion protein where antibodies against TUBA1A and GFP were used as absorbing agents, considering that TUBB4A antibody for IP was commercially unavailable. We again detected the interaction of *TubAR* with TUBA1A and TUBB4A (Fig. 1i, j). Serving as a negative control, lncRNA *NEAT1* showed no interaction with TUBA1A or TUBB4A (Fig. 1i, j). Thus, the lncRNA *TubAR* is a tubulin-associated cytoplasmic RNA, and is expressed in Purkinje cells and oligodendrocytes of the mouse cerebellum.

### *TubAR* forms a ternary complex with TUBB4A–TUBA1A heterodimer and promotes the TUBB4A–TUBA1A interaction

**Fig. 2:**
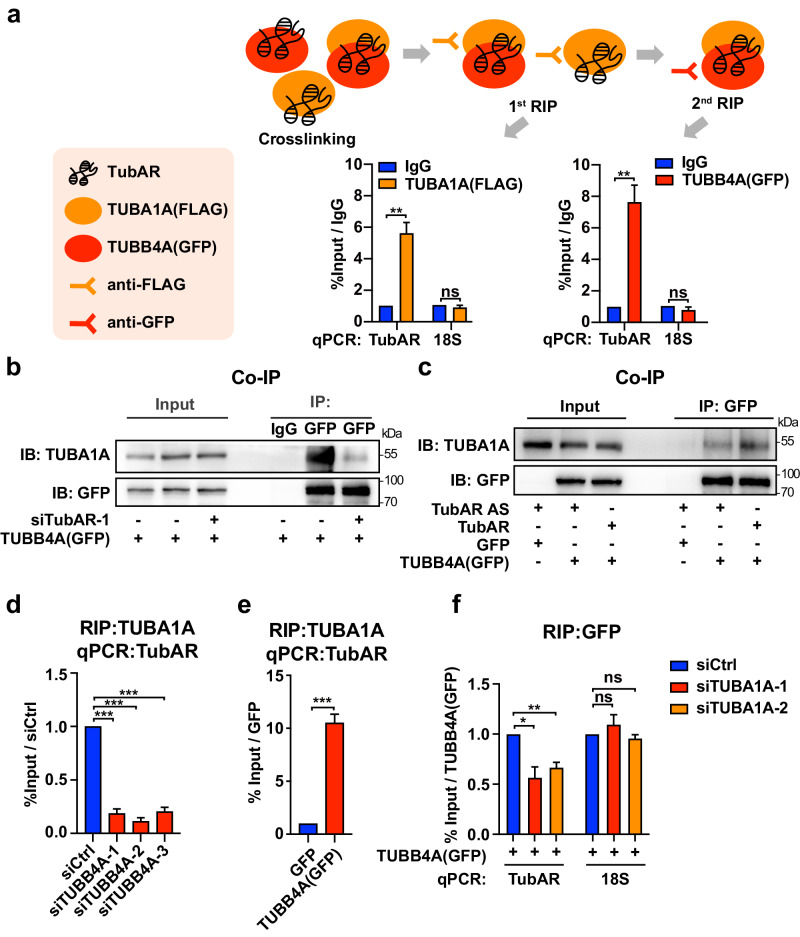
*TubAR* forms an RNA-protein complex with TUBB4A/TUBA1A. **a** Upper: Illustration flow-chart of two round RIP assay. Lower: Two round RIP assay showing *TubAR* simultaneously interacts with FLAG-TUBA1A and GFP-TUBB4A (*n* = 3). IgG, negative antibody control. *18* *S*, negative RNA control. **b** Co-IP showing decreased interaction of TUBB4A–TUBA1A in Neuro-2a cell extracts expressing si*TubAR*-1. IgG, negative antibody control. **c** Co-IP showing increased interaction of TUBB4A–TUBA1A in HeLa cell extracts expressing *TubAR*. GFP, negative antibody control. **d** RIP assays showing decreased interaction of *TubAR*–TUBA1A in Neuro-2a cells expressing the indicated siTUBB4As (*n* = 3). **e** RIP assays showing increased interaction of *TubAR*–TUBA1A in Neuro-2a cells expressing GFP-TUBB4A (*n* = 3). **f** RIP assays showing the decreased interaction of *TubAR*–TUBB4A in Neuro-2a cells expressing indicated siTUBA1As (*n* = 3). Data are represented as means ± SEM. Statistical significance was determined using unpaired *t*-test with Welch’s correction; ns, *P* > 0.05; *P < 0.05; **P < 0.01; ***P < 0.001.

As *TubAR* can interact with both TUBB4A and TUBA1A, we hypothesized that the three components may form a ternary protein–RNA complex. To test that, we performed sequential RIP analysis for TUBA1A and TUBB4A, followed by quantitative polymerase chain reaction (qPCR) analysis for *TubAR*. Using Neuro-2a cells co-expressing FLAG-TUBA1A and GFP-TUBB4A, our results showed existence of the ternary TUBB4A–*TubAR*–TUBA1A complex (Fig. 2a).

To investigate the potential role of *TubAR* in the TUBB4A–TUBA1A interaction, we knocked down *TubAR* in Neuro-2a cells, and observed dramatic decrease of the interaction between GFP-TUBB4A and TUBA1A (Fig. 2b). HeLa cells have been extensively used in studies of microtubule dynamics^46,48^ and do not express *TubAR*. Ectopic expression of *TubAR* in HeLa cells caused an obvious increase of TUBB4A–TUBA1A interaction (Fig. 2c). Consistent with our results in mouse cerebellum (Fig. 1h; Supplementary Table S1), RNA pull-down assay showed that *TubAR* is able to pull down TUBA1A and TUBB4A from HeLa cells as well (Supplementary Table S2). When we included non-biotinylated *TubAR* in pull-down assay to compete with biotinylated-*TubAR* for target protein bindings, a reduction in the enrichment of TUBA1A and TUBB4A was observed (Supplementary Fig. S3).

Moreover, we found that modulation of TUBB4A by siRNA or GFP-TUBB4A expression had a positive effect on the TUBA1A–*TubAR* interaction (Fig. 2d, e), and knockdown of TUBA1A by siRNA could also significantly decrease the TUBB4A–*TubAR* interaction (Fig. 2f). Note that *TubAR* knockdown did not affect TUBA1A or TUBB4A level in Neuro-2a cells (Supplementary Fig. S4a), and that knockdown of TUBB4A or TUBA1A did not affect *TubAR* level (Supplementary Fig. S4b, c). These results suggest that lncRNA *TubAR* is an essential modulator for promoting the αβ-tubulin heterodimer formation in mammalian cells.

### *TubAR* promotes microtubule assembly

**Fig. 3:**
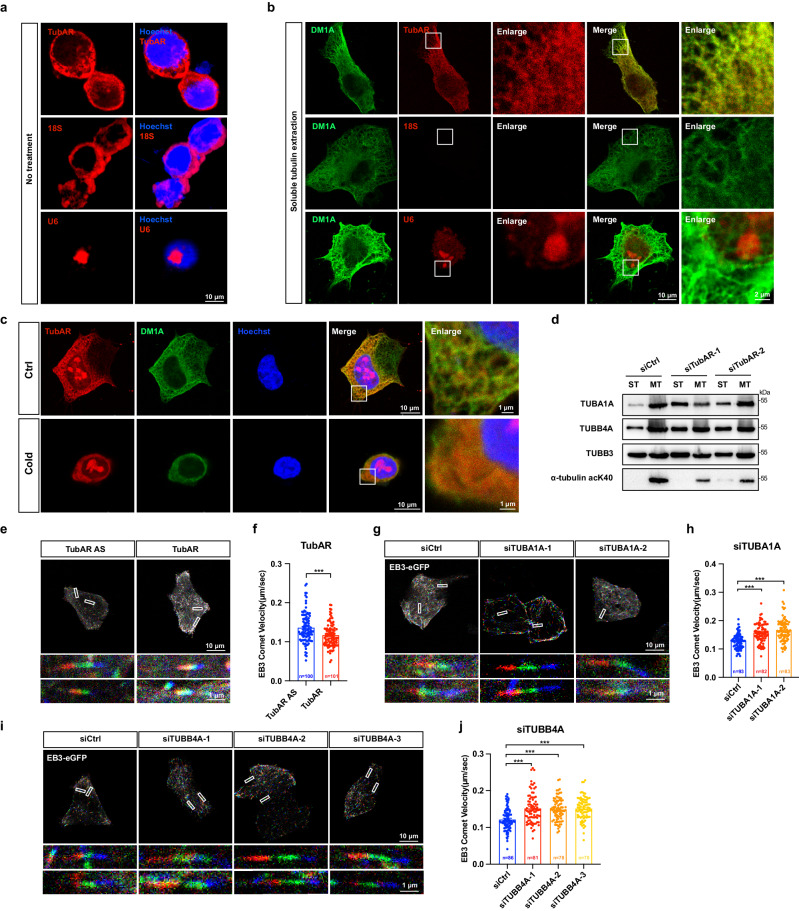
*TubAR* promotes microtubule assembly. **a** FISH of *TubAR*, *18* *S*, and *U6* in untreated Neuro-2a cells. **b** Immuno-FISH of *TubAR*, *18* *S*, and *U6* with α-tubulin in Neuro-2a cells after soluble tubulin extraction, showing that *TubAR* co-localizes with microtubules. **c** Immuno-FISH of *TubAR* in cold-treated Neuro-2a cells. **d** Distribution of TUBA1A, TUBB4A, and TUBB3 in soluble tubulin fraction or microtubule fraction in Neuro-2a cells expressing si*TubAR*s or siCtrl. Acetylation of K40 in α-tubulin (α-tubulin acK40) serves as a marker for microtubule fraction^66^. **e**, **g**, **i** Live imaging of EB3 comet in HeLa cells expressing *TubAR* (**e**), siTUBA1A (**g**), and siTUBB4A (**i**) visualized by viewing successive frames as different channels, red, green, and blue, creating a rainbow effect. More rapid polymerizations result in a longer rainbow with less overlap between the colors and slower polymerizations result in a shorter rainbow with greater overlap in the colors manifested as white. The images were pooled from plus-end microtubule binding assay. Representative videos of EB3 tracking behavior can be seen in Supplementary movies. **f**, **h**, **j** Quantification of EB3 comet velocity of *TubAR* (**f**), TUBA1A (**h**, knockdown), and TUBB4A (**j**, knockdown) on the microtubule assembly dynamics in HeLa cells. *TubAR* antisense (*TubAR* *AS*), serving as a negative control to *TubAR*; n number of cells pooled from three independent experiments. Data are represented as means ± SEM. Statistical significance was determined using unpaired *t*-test with Welch’s correction (**f**, **h**, **j**); ***P < 0.001.

Next, we performed immuno-FISH to assess whether *TubAR* colocalizes with microtubules in Neuro-2a cells. Compared to the untreated cells (Fig. 3a), *TubAR*, but not the control *18* *S* and *U6* RNAs, presented a clear co-localization with microtubules when free soluble tubulins were extracted (Fig. 3b). To further validate such co-localization, we conducted a classic cold-induced microtubule depolymerization assay with Neuro-2a cells. When the cells were exposed to cold treatment, immunostaining revealed an almost complete disruption of microtubule network and the signal of *TubAR* was also disrupted (Fig. 3c), confirming the co-localization of *TubAR* with microtubule in cells.

Furthermore, we conducted co-sedimentation assay to test interaction of *TubAR* and microtubules. We found that >80% of *TubAR* was distributed in the microtubule-containing pellet after super centrifugation (Supplementary Fig. S5a, b). In sharp contrast, almost all the *TubAR* *AS* (a complimentary sequence to *TubAR*, serving as a negative control) was detected in the supernatant fraction (Supplementary Fig. S5a, b). Reactions without tubulins showed that neither *TubAR* nor *TubAR* *AS* was able to be precipitated (Supplementary Fig. S5a, b), eliminating the possibility of *TubAR* precipitation other than interacting with microtubule.

To test whether *TubAR* affects microtubule assembly, we knocked down *TubAR* in Neuro-2a cells and detected TUBA1A and TUBB4A levels in cell fractions containing soluble αβ-tubulin heterodimers (ST) or insoluble microtubules (MT). We found that relative levels of both TUBA1A and TUBB4A in the soluble fraction were dramatically increased in the presence of si*TubARs* (Fig. 3d). In contrast, TUBB3, a tubulin isotype that shows no interaction with *TubAR* and serves as a negative control in this experiment, exhibited similar distributions in both fractions under the treatment of si*TubAR*s (Fig. 3d). Thus, our data suggest that *TubAR* possesses a positive ability to promote soluble αβ-tubulin heterodimers to be assembled into microtubules.

To investigate effect of *TubAR* on microtubule dynamics, we monitored microtubule growth by tracking plus-end-binding protein in live HeLa cells. By using GFP-labeled EB3 for visualization, we showed that ectopically expressed *TubAR* can promote the assembly of microtubules at a slower growth rate than control RNA (*TubAR* *AS*) (Fig. 3e, f; Supplementary Video S1). Consistently, suppression of TUBA1A (Fig. 3g, h; Supplementary Video S2) or TUBB4A (Fig. 3i, j; Supplementary Video S3) by siRNAs significantly accelerated growth rate. These interesting findings are in line with previous observations that either TUBA1A- or TUBB4A-composed microtubules exhibit slower growth rates than other tested isotype-composed microtubules^22,23^. Thus, our data suggest that *TubAR* can facilitate the assembly of those microtubules that are specifically composed of the cerebellar-prevalent TUBB4A and TUBA1A.

### Two groups of TUBB4A mutants exhibit distinct binding capacities towards *TubAR* and TUBA1A

**Fig. 4:**
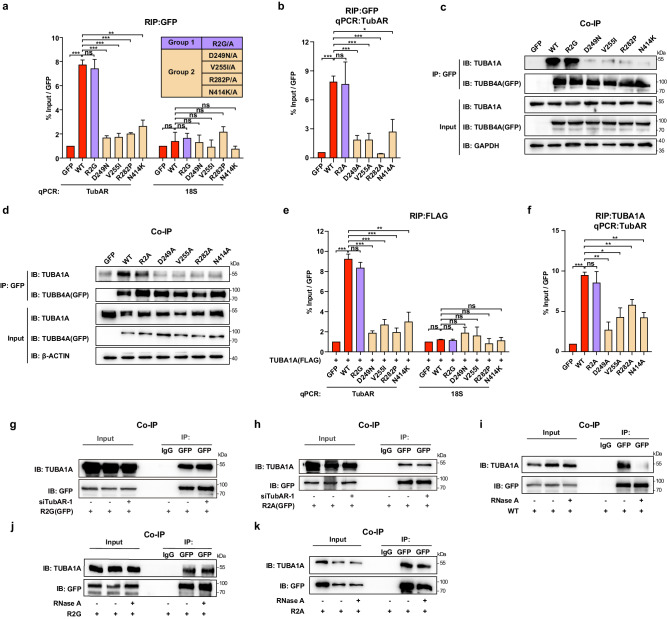
Two groups of TUBB4A mutations exhibit distinct *TubAR* and TUBA1A binding capacities. **a** Upper right: Illustration of the two groups of disease-related TUBB4A variants. Group 1 contains one reported non-hypomyelination-causing variant. Group 2 contains four reported hypomyelination-causing variants. Lower left: RIP assays showing distinct binding capacities of GFP-TUBB4A wild-type (WT) or the indicated mutations with *TubAR*. (*n* = 3). *18* *S*, a negative lncRNA control. **b** RIP assays showing interaction of *TubAR*–TUBB4A in Neuro-2a cells expressing GFP-tagged TUBB4A WT or the indicated variants (*n* = 3). **c**, **d** Co-IP to assess interaction changes of TUBA1A and GFP-tagged TUBB4A WT or the indicated variants in Neuro-2a transfectants. GAPDH and β-actin, loading control. **e**, **f** RIP assays showing the interaction of *TubAR*–TUBA1A in Neuro-2a cells expressing GFP-tagged TUBB4A WT or the indicated variants (*n* = 3). *18* *S*, negative lncRNA control. **g**, **h** Co-IP to assess interaction of TUBA1A with GFP-TUBB4A-R2G (**g**) or GFP-TUBB4A-R2A (**h**) in Neuro-2a cells expressing si*TubAR*-1. IgG, negative antibody control. **i**–**k** Co-IP to assess interaction changes of TUBA1A with GFP-TUBB4A WT (**i**), GFP-TUBB4A-R2G (**j**), or GFP-TUBB4A-R2A (**k**) in Neuro-2a cell extracts treated with RNase A. IgG, negative antibody control. Data are represented as means ± SEM. Statistical significance was determined using unpaired *t*-test with Welch’s correction; ns, *P* > 0.05; *P < 0.05; **P < 0.01; ***P < 0.001.

As aforementioned, the hypomyelination-causing mutations of TUBB4A (D249N, V255I, R282P, and N414K) alter microtubule assembly, whereas normal microtubule assembly occurs with the non-hypomyelination-causing mutation R2G^46^. We next investigated whether these TUBB4A mutations have differential binding capacity to *TubAR*. Our RIP results showed that, compared to wild-type GFP-TUBB4A, *TubAR* binding capacities were remarkably reduced for all of the variants with known hypomyelination-causing mutations (Fig. 4a). In sharp contrast, non-hypomyelination-causing mutation R2G exhibited full binding capacity to *TubAR*, as that of the wild-type GFP-TUBB4A (Fig. 4a). Similar binding capacities were also detected when the tested residues were substituted to alanine (Fig. 4b).

We also tested binding capacities between these TUBB4A variants and TUBA1A. Co-IP showed that binding capacities of all hypomyelination-causing variants of TUBB4A to TUBA1A were remarkably reduced (Fig. 4c, d), while R2G/A retained full binding capacity to TUBA1A as that of wild-type GFP-TUBB4A (Fig. 4c, d). In agreement with a report conducted with HEK293 cells^46^, similar protein levels of GFP-tagged TUBB4A constructs in Neuro-2a transfectants and TUBA1A protein levels were detected (Fig. 4c, d), ruling out the possibility that the decreased interaction could have resulted from different levels of the TUBB4A mutations or altered levels of the TUBA1A protein. Thus, the distinct binding ability to TUBA1A of these two TUBB4A variant groups provides a molecular mechanism for their reported different impacts on microtubule assembly and hypomyelination phenotype of the patients.

We next examined impacts of these two TUBB4A variant groups on interaction of *TubAR* with TUBA1A. An RIP assay showed similar binding capacities to wild-type GFP-TUBB4A and the R2G/A mutants (Fig. 4e, f), whereas significantly reduced signals were observed for all the hypomyelination-causing mutations (Fig. 4e, f). These results indicate that both wild-type TUBB4A and non-hypomyelination-causing variants R2G/A possess similar interaction capacities for *TubAR* and TUBA1A.

Recall that TUBB4A–TUBA1A heterodimer formation requires *TubAR* (Fig. 2b), we then tested effect of *TubAR* on TUBA1A’s interaction with the R2G/A mutations. Surprisingly but intriguingly, we found that the *TubAR* knockdown did not disrupt the interaction of R2G/A to TUBA1A (Fig. 4g, h), suggesting a gained constitutive interaction of the R2G/A mutations with TUBA1A in a *TubAR*-independent manner. Similar results as those for si*TubAR* treatments were observed when we treated the cell lysates with RNase A for wild-type TUBB4A and R2G/A mutations (Fig. 4i–k). These results further support our previous observation that *TubAR* is required for the TUBB4A–TUBA1A heterodimer formation (Fig. 2b).

### Restored TUBB4A–TUBA1A heterodimers rescue *TubAR* knockdown-triggered cell death

**Fig. 5:**
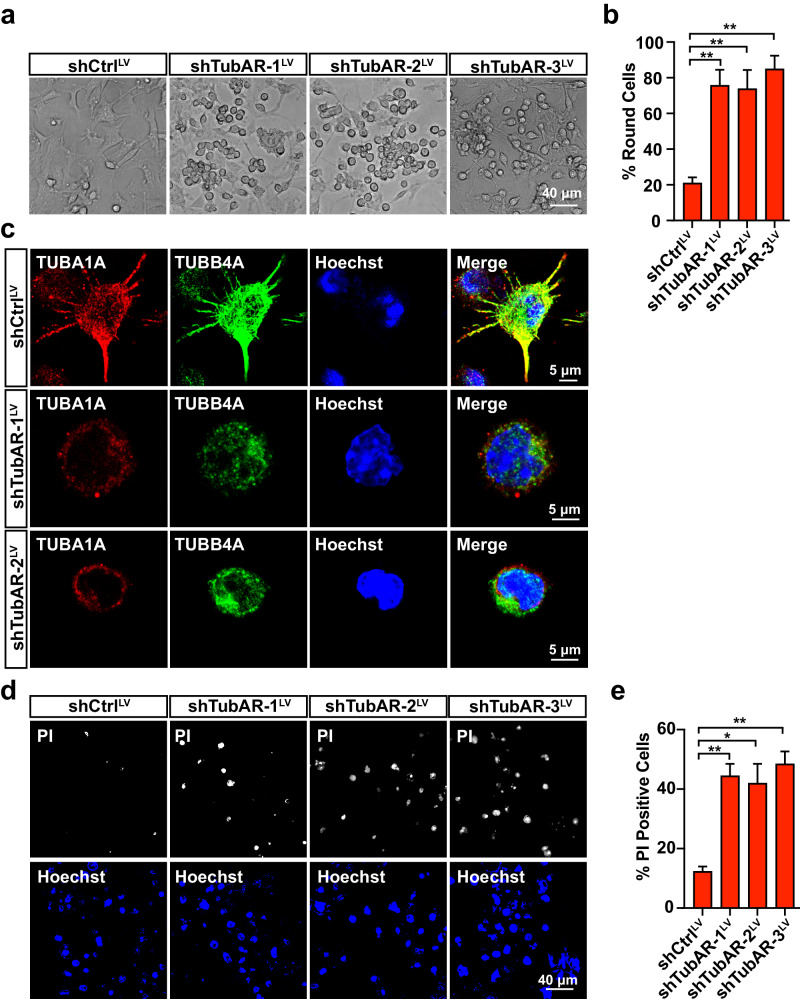
Knockdown of *TubAR* causes abnormal cell shape, and increased cell death in Oli-neu cells. **a** Representative images of Oli-neu cells infected with the shCtrl^LV^ or the indicated sh*TubAR*s^LV^ at 36 h post infection. **b** Quantification of the round cells in **a** (*n* = 3). **c** Representative images for immunostaining of TUBA1A or TUBB4A in the shCtrl^LV^ or the indicated sh*TubAR*s^LV^ infected Oli-neu cells at 24 h post infection. **d** Representative images of PI staining of the Oli-neu cells infected with the shCtrl^LV^ or the indicated sh*TubAR*s^LV^ at 24 h post infection. **e** Quantification of PI-positive cells in **d** (*n* = 3). Data are represented as means ± SEM. Statistical significance was determined using unpaired *t*-test with Welch’s correction; ns, *P* > 0.05; *P < 0.05; **P < 0.01. LV lentivirus.

**Fig. 6:**
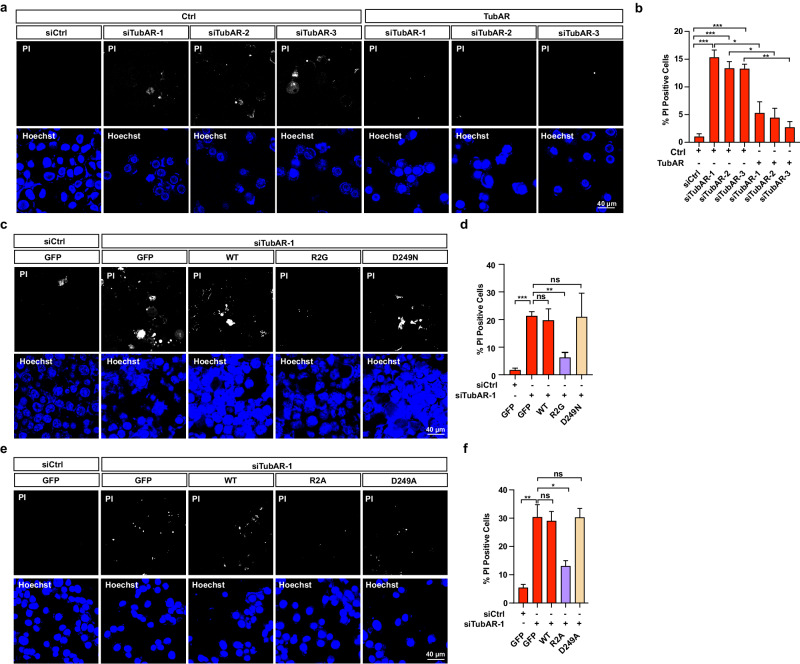
TUBB4A-R2A/G variants rescue *TubAR* knockdown-induced cell death in Neuro-2a cells. **a** PI staining in the *TubAR* knocked-down Neuro-2a cells at 48 h post infection. **c**, **e** PI staining evaluating effects of GFP-tagged TUBB4A WT or the indicated variants in *TubAR* knocked-down Neuro-2a cells at 48 h post infection. **b**, **d**, **f** Quantification of cell viability (assessed as PI-positive cells) in **a**, **c**, **e** (*n* = 3). Data are represented as means ± SEM. Statistical significance was determined using unpaired *t*-test with Welch’s correction; ns, *P* > 0.05; *P < 0.05; **P < 0.01; ***P < 0.001.

To investigate biological function of *TubAR*, we knocked down *TubAR* by infecting three independent lentivirus-packaged sh*TubAR*s in Oli-neu cells, and made a striking observation of aberrant round morphology (Fig. 5a–c). Staining of these *TubAR* knocked-down Oli-neu cells with Propidium Iodide (PI) revealed that *TubAR* knockdown induced severe cell death (Fig. 5d, e). Similar cell death triggered by *TubAR* knockdown was also observed in Neuro-2a cells (Fig. 6a, b). The *TubAR* knockdown-triggered cell death could be rescued upon transfecting cells with *TubAR*-expressing plasmid (Fig. 6a, b). Thus, *TubAR* is indispensable for cell survival.

To test whether the observed cell death results from disruption of TUBB4A–TUBA1A interaction, we took advantage of the unique feature of the TUBB4A–R2G/A variants, which have gained the ability to constitutively bind to TUBA1A in an RNA-independent manner. We co-transfected Neuro-2a cells with the TUBB4A variants bearing R2G or R2A mutation in the presence of si*TubAR*-1, and found that both R2G and R2A could reverse si*TubAR*-1-triggered cell death (Fig. 6c–f). In contrast, the wild-type GFP-TUBB4A (interacting with TUBA1A in an RNA-dependent manner) or the D249N/A variants (unable to interact with TUBA1A) failed to rescue the si*TubAR*-1-triggered cell death (Fig. 6c–f). These results support that cell death induced by *TubAR* knockdown results from disruption of TUBB4A–TUBA1A heterodimer formation.

### Knockdown of *TubAR* in mouse cerebellum results in demyelination and disrupted integrity of oligodendrocytes and Purkinje cells

Next, we sought to delineate whether disrupted TUBB4A–*TubAR*–TUBA1A RNA–protein complex would affect physiological functions. Seeking to avoid potential interference from DNA cis-acting elements and the overlapped RNAs with *TubAR* (Supplementary Fig. S1a), we designed a *TubAR*-specific knockdown strategy which achieved an ~50% knockdown efficiency without affecting the neighboring genes (Supplementary Fig. S6). Fluorescence images of EGFP were taken to characterize the successfully infected brain areas: for both the shCtrl^AAV^- and sh*TubAR*s^AAV^-injected mice, strong EGFP signals were evident in the white matter of the cerebellum vermis and in the lobular cortex near the injection sites (Supplementary Fig. S7a, b, d).

**Fig. 7:**
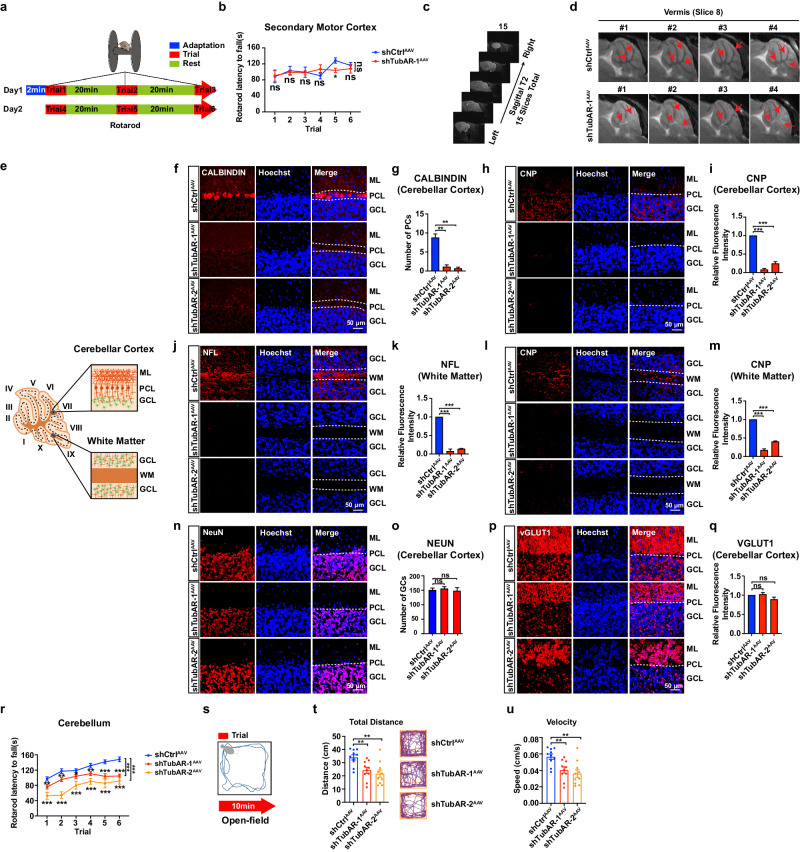
Cerebellum-specific knockdown of *TubAR* results in reduced numbers of oligodendrocytes and Purkinje cells, demyelination, and decreased locomotor activity. **a** Illustration of rotarod behavioral test shown in **b**, **k**. **b** Rotarod test results from shCtrl^AAV^ (*n* = 5) and sh*TubAR*-1^AAV^ (*n* = 5) secondary motor cortex-injected mice. **c** Schematic illustration of MRI analysis. **d** MRI images of Slice 8 (vermis) in four shCtrl^AAV^ and four sh*TubAR*-1^AAV^ mice. Arrows indicate areas with altered MRI signals between shCtrl^AAV^ and sh*TubAR*-1^AAV^ mice. **e** Schematic illustration of the microscopic analytic region for **f**–**q**. **f**, **h**, **n**, **p** Representative immunostaining images of CALBINDIN (**f**), CNP (**h**), NeuN (**n**), and vGLUT1 (**p**) in the shCtrl^AAV^ and sh*TubAR*s^AAV^ mice at cerebellar cortex region. Slice thickness, 40 µm. **g**, **i**, **o**, **q** Quantification of CALBINDIN, CNP, NeuN, and vGLUT1 using ImageJ in **f**, **h**, **n**, **p** (*n* = 3). **j**, **l** Representative immunostaining images of NFL (**j**) and CNP (**l**) in the shCtrl^AAV^ and sh*TubAR*s^AAV^ mice at white matter region. Slice thickness, 40 µm. **k**, **m** Quantification of NFL and CNP using ImageJ in **j**, **l** (*n* = 3). **r** Rotarod test results from shCtrl^AAV^ (*n* = 36), sh*TubAR*-1^AAV^ (*n* = 39) and sh*TubAR*-2^AAV^ (*n* = 13) cerebellum-injected mice. **s** Illustration of open-field behavioral test shown in **t**, **u**. **t**, **u** Total movement distance (**t**, left), representative recorded routes (**t**, right) and velocity of active movement (**u**) of open-field test from shCtrl^AAV^ (*n* = 10), sh*TubAR*-1^AAV^ (*n* = 10), and sh*TubAR*-2^AAV^ (*n* = 10) cerebellum-injected mice. Data are represented as means ± SEM. Statistical significance was determined using unpaired *t*-test with Welch’s correction (**g**, **i**, **k**, **m**, **o**, **q**) or mixed-effects model with Geisser–Greenhouse correction (**b**, **r**); ns, *P* > 0.05; *P < 0.05; **P < 0.01; ***P < 0.001. AAV adeno-associated virus, ML molecular layer, PCL Purkinje cell layer, GCL granule cell layer, WM white matter, PC Purkinje cell.

Through magnetic resonance imaging (MRI), we found that all four tested sh*TubAR*^AAV^-1 mice exhibited demyelination, as indicated by higher density signals in the white matter of the cerebellar vermis, as compared to the tested shCtrl^AAV^ mice (Fig. 7c, d; Slices 4–10 in Supplementary Fig. S8a, b). These results indicate that the sh*TubAR*^AAV^ mice have cerebellar myelin deficiencies that are apparently similar to those of human patients carrying hypomyelination-causing TUBB4A variants^46^.

In the cerebellum, Purkinje cells give rise to the sole output of the cerebellar cortex. The axons of Purkinje cell pass through the granule cell layer to the white matter and form fasciculated and intensively myelinated fibers that are covered by oligodendrocytes. Recalling that *TubAR* is expressed in both Purkinje cells and oligodendrocytes in cerebellar white matter (Fig. 1f), we examined potential changes in Purkinje cells and oligodendrocytes in sh*TubAR*s^AAV^ mice using immunofluorescent staining against CALBINDIN, Neurofilament Light Chain (NFL), and CNP. Note that NFL is a marker for myelinated axons and mainly recognizes Purkinje cell axons in cerebellar white matter. Strong CALBINDIN, NFL, and CNP signals were detected in the shCtrl^AAV^ mice (Fig. 7e–m). In contrast, these signals were dramatically decreased (to background levels) in both sh*TubAR*^AAV^-1 and sh*TubAR*^AAV^-2 mice (Fig. 7e–m). These findings indicate that disruption of the TUBA1A–*TubAR*–TUBB4A ternary complex results in demyelination and disrupted integrity of oligodendrocytes and Purkinje cells.

In order to confirm results of CNP, NFL, and CALBINDIN staining, we were seeking other markers to be negative controls. Neuronal Nuclei (NeuN) is a marker for mature neurons. In cerebellum, it exclusively labels granule cells. Immunofluorescent staining against NeuN showed no difference between the sh*TubAR*s^AAV^ and shCtrl^AAV^ mice (Fig. 7n, o). Parallel fibers are axonal projections that extend from granule cells in the cerebellar cortex and have been known as non-myelinated nerve fibers^49^. Immunofluorescent staining against Vesicular Glutamate Transporter 1 (vGLUT1, a commonly used marker for parallel fibers) on the mouse cerebellar sections showed no difference in the intensity of parallel fibers for sh*TubAR*s^AAV^ and shCtrl^AAV^ mice (Fig. 7p, q).

### *TubAR* knockdown mice display decreased locomotor activity

The secondary motor cortex (M2) receives sensory information and maintains a flexible mapping diagram of sensorimotor associations in the service of adaptive choice behavior in both mice and humans^50^. Considering the barely detectable level of *TubAR* in the M2 region (Fig. 1a), we reasoned (and later experimentally confirmed) that injection of sh*TubAR*^AAV^ into the M2 would not disrupt normal motor function, thus serving as a suitable negative control to mice received injection in the cerebellum for behavioral analysis (Fig. 7a, b; Supplementary Fig. S7a, c).

Consistent with all of the above identified myelin and neuronal defects in the sh*TubAR*s^AAV^ mice, rotarod tests and open-field tests revealed decreased locomotor activities in sh*TubAR*s^AAV^ mice, indicated by the measurements on latency to fall (Fig. 7r), total movement distance (Fig. 7s, t), and velocity of active movement (Fig. 7s, u), compared to the shCtrl^AAV^ mice. Based on our results, it is plausible that the disruption of microtubule assembly is the basis of cerebellar malfunctions displayed in the sh*TubAR*s^AAV^ mice.

## Discussion

In this study, we have discovered that *TubAR*, a cerebellum-enriched lncRNA, is the long-elusive structural RNA that functions in cytoskeleton assembly. Further, we have elucidated the molecular mechanism through which *TubAR* interacts with and mediates TUBB4A and TUBA1A heterodimer formation, as well as promotes TUBB4A/TUBA1A-composed microtubule assembly. We have also demonstrated how these biomolecular events manifest as emergent consequences in neuron/oligodendrocyte survival, myelination, and ultimately in the motor-regulatory activities of the mammalian cerebellum.

It has been well known that microtubule assembly is tightly regulated by tubulin isotypes, microtubule-associated factors and the presence of diverse post translational modifications on these protein factors^22,23,48,51–55^. From 1977 to 2009, four studies showed that centrosome and spindle structure were greatly impaired by RNase treatment^2–5^, suggesting a critical role of RNA in supporting the structural integrity of the microtubule-based cytoskeleton. Recent advancements in technologies such as deep sequencing and cross-linking immunoprecipitation have allowed for the identification of numerous RNAs associated with microtubule-based structures^56–58^. However, the currently reported RNAs on microtubules are primarily involved in local translation or trafficking purposes^57,59–61^. In the present work, we show that *TubAR* is required for the TUBA1A/TUBB4A–tubulin heterodimer formation, and can promote the soluble TUBA1A/TUBB4A-tubulin heterodimers to be further incorporated into microtubules. Identification of *TubAR*, to the best of our knowledge, provides the first structural RNA to support the long-standing hypothesis that RNA can function as a direct organizer of cytoskeleton structure.

In the present work, we identify that the TUBB4A variants at p.Arg2 residue (R2G and R2A) exhibit different features from those hypomyelination-causing TUBB4A variants: they gain the ability to interact with TUBA1A in an RNA-independent manner, although they themselves retain the ability to interact with *TubAR*. Discovery of these unique features provides a valuable tool for our study and sheds light on how α- and β-tubulin interact. Firstly, by utilizing these features, we can introduce αβ-tubulin heterodimers (by transfecting R2G/A mutations) into those cells that are simultaneously underwent *TubAR* siRNA treatment (effectively disrupting the tubulin heterodimers), and thus establishing the requirement of *TubAR*’s biological function in proper αβ-tubulin heterodimer formation. Secondly, these identified features of the R2G mutation coincide with its previously reported functional normalities in oligodendrocyte morphology and microtubule assembly, which provides a molecular answer to how patients carrying R2G mutation do not exhibit demyelination. Finally, these identified features of R2G mutation suggest that TUBB4A interacts with *TubAR* and TUBA1A through distinct sites or domains. Taking into account our other biochemical findings, a two-step model is proposed to reconcile our findings. In this model, we propose that the wild-type TUBB4A first recognizes *TubAR*, and this interaction induces a further conformational change on TUBB4A, so that TUBB4A acquires binding capacity to TUBA1A.

In summary, the present study allows us to look beyond the previously reported functions of lncRNAs in gene regulation and signal transduction. The identification of *TubAR* provides the first structural RNA individual to support the long-standing hypothesis that RNA can act as a direct organizer of cytoskeletal structure, and thus represents a profound departure from the current understanding of how regulatory RNAs exert their biological roles in cells. As we start to learn about the structural requirements of lncRNA in microtubule assembly, we may need to think of redefining our understanding of cell structure formation.

## Materials and methods

### Plasmids

To generate GFP-tagged full-length mouse TUBB4A and its site-specific mutations, PCR-amplified TUBB4A cDNA was cloned into the pEGFP-C1 vector with EcoRI and KpnI digestion. Site-specific mutations of TUBB4A were generated by PCR-based, site-directed mutagenesis kit (Vazyme, Cat#C215-01) according to the manufacturer’s instructions. To generate FLAG-tagged full-length mouse TUBA1A, PCR-amplified TUBA1A cDNA was cloned into the 3× FLAG-Myc-CMV-24 vector with HindIII and BamHI digestion. To generate EGFP-tagged full-length mouse EB3, PCR-amplified EB3 cDNA was clone into the EGFP-N1 vector by PCR-based, One Step Cloning Kit (Vazyme, Cat#C112-01). To generate *TubAR* in vitro transcription template, PCR-amplified *TubAR* cDNA (4623–7363 nt) was cloned into the pcDNA3.1(+) vector with XhoI and EcoRI digestion. Complimentary sequence to *TubAR* (*TubAR AS*, complementary sequence to 7363–4623 nt) was also cloned into the pcDNA3.1(+) vector and served as a negative control.

### Cell culture, differentiation, and transfection

Oli-neu cells were maintained in DMEM/F12 with 2% B27 (Gibco, Cat#17504044), 5% fetal bovine serum (FBS, Viva Cell, Shanghai, China, Cat#C04001-500) and 2% horse serum (Viva Cell, Shanghai, China, Cat#C2510-0100). The Neuro-2a cells, HeLa cells and the HEK293T cells were grown in DMEM (Viva Cell, Shanghai, China, Cat#C3113-0500) supplemented with 10% FBS. For differentiation, Neuro-2a cells were cultured in DMEM/opti-MEM (Gibco, Cat#31985070; 1:1) for 72 h.

SiRNAs were designed and synthesized by Ribobio. Cultured cells were transfected at a confluency of 1 × 10^6^ cells with 5 μg plasmid or 150 pmol siRNA using Lipofectamine 3000 Transfection Reagent (Invitrogen, Cat#L3000015; Neuro-2a cells and HEK293T cells) or HieffTrans® Universal Transfection Reagent (Yeason, Cat#40808ES03; HeLa cells) according to the manufacturer’s instructions. Transfectants were harvested 48 h post transfection unless otherwise noted, followed by the desired experiments.

The siRNA sequences used are as follows: si*TubAR-1*: TCAATTCCTTGCATTGCAT; si*TubAR-2*: GGAGGGCCAAGAGAAGTTT; si*TubAR-3*: GCTCATCCTGTGGTCAAAT.

### Lentivirus packaging and infection

HEK293T cells grown on a 6-cm dish were transfected with 2 μg of PLKO.1, 2 μg of pRev, 2 μg of pGag/Pol, and 1 μg of pVsvg using Lipofectamine 3000 Transfection Reagent. The medium was changed at 24 h post transfection. At 48 h post transfection, the supernatants containing the packaged lentivirus were collected and then stored at –80 °C for future infection. Infection was conducted when the target cells reached to 60%–70% confluency. The lentivirus packaged shRNA sequences used are as follows: sh*TubAR-1*: GCATAATTCTCGGTCTGCA; sh*TubAR-2*: GACACGTCTTAGTTGTTCA; sh*TubAR-3*: CGAACCAAGATGTACGTGT.

### Western blotting assay, separation of soluble tubulin and microtubule

Western blotting assay was performed as described previously^62^. GAPDH or β-ACTIN protein was used as a loading control. The primary antibodies used were GAPDH (Proteintech, Cat#60004-1-Ig, 1:5000), β-ACTIN (Proteintech, Cat#66009-1-Ig, 1:5000), GFP (Proteintech, Cat#50430-2-AP, RRID: AB_11042881, 1:5000), TUBA1A (Sigma Aldrich, Cat#HPA043684, RRID: AB_2678617, 1:1000), TUBB4A (Abcam, Cat#ab11315, RRID: AB_297919, 1:1000), and FLAG (Abcam, Cat#ab125243, RRID: AB_11001232, 1:2000). The secondary antibodies used were Goat anti-Mouse (Proteintech, Cat#SA00001-1, 1:5000) or Goat anti-Rabbit (Proteintech, Cat#SA00001-2, 1:5000). Immunoreactive proteins were visualized using the Tanon chemiluminescence gel imaging system (Tanon 5200Muti).

As described previously^63^, for the separation of soluble tubulin and microtubules, cells were washed once with PBS and PHEM buffer, respectively, and resuspended in PHEM buffer containing 0.1% Triton X-100 and 0.1% DMSO at RT for 10 min. The cells were then centrifugated at 12,000 rpm for 10 min. The supernatant fraction was collected as soluble tubulin fraction. The cell pellet was then resuspended in PHEM buffer containing 0.1% Triton X-100 and 0.1% DMSO again for 10 min and centrifugated at 12,000 rpm for 10 min. The supernatant was collected as microtubule fraction. The collected fractions were subjected for immunofluorescence or western blotting assay.

### RNA isolation, reverse transcription and qPCR

RNA isolation and qPCR were performed as described previously^62^. The primers used for the real-time PCR are listed below.

*Gapdh*:

Forward primer: 5′-CATGGCCTTCCGTGTTCCT-3′;

Reverse primer: 5′-TGATGTCATCATACTTGGCAGGTT-3′;

*TubAR*:

Forward primer: 5′-GAGCAGGTAAGTGGCTTGGT-3′;

Reverse primer: 5′-TTTGCTTGGGCTCTCACCTC-3′;

*18* *S*:

Forward primer: 5′-GCCGCGGTAATTCCAGCTCCAA-3′;

Reverse primer: 5′-GCTCGGGCCTGCTTTGAACACT-3′;

*NEAT1*:

Forward primer: 5′-AGGAGAAGCGGGGCTAAGTA-3′;

Reverse primer: 5′-TAGGACACTGCCCCCATGTA-3′;

*Tuba1a*:

Forward primer: 5′-TGGGAGGGTGTCTTGGTATCT-3′;

Reverse primer: 5′-CCGTAATCCACAGAGAGCCG-3′;

*Tubb4a*:

Forward primer: 5′-CTGGGACCTATCATGGGGAC-3′;

Reverse primer: 5′-CCTGCTCCGGATTGACCAAA -3′.

### RACE

3′ RACE System (Invitrogen, Cat#18373-019) and 5′ RACE System (Invitrogen, Cat#18374-058) were performed according to the manufacturer’s instructions.

For 3′ RACE, total RNAs from cerebellum were converted into cDNA using reverse transcriptase (RT) with an oligo-dT adapter primer. Specific cDNA was then amplified by PCR using a gene-specific primer (GSP1) that annealed to a region of known exon sequence and an adapter primer that targeted the poly(A) tail region. Nested PCR was used to improve the specificity of products with GSP2.

For 5′ RACE, an antisense gene-specific primer (GSP1) was used for the synthesis of specific cDNA by reverse transcriptase. Prior to PCR, an adapter sequence was annealed to the unknown 5′-sequences of the cDNA in a TdT-tailing step. Specific cDNA was then amplified by PCR using GSP2 that annealed in a region of known exon sequence and an adapter primer that targeted the 5′ terminus. Then the nested PCR was carried out using GSP3. The final products of RACE experiments were recovered by agarose electrophoresis and sent for Sanger sequencing. The primers used are as follows:

For 3′ RACE:

*GSP1*: 5′-GAGGCAGGGGGACAGAAATTTCA-3′;

*GSP2*: 5′-GGAGGAGAGAGAAGCTAGAGACG-3′;

For 5′ RACE:

*GSP1*: 5′-GCATTTTATTCACCGT-3′;

*GSP2*: 5′-CCAAGGTGTATGGGCAAAAGA-3′;

*GSP3*: 5′-CCAAGGTGCATGGTCAAGT-3′.

### PI/Hoechst 33342 staining

To detect the Oli-neu cell or Neuro-2a cell death, PI/hoeschst staining (Solarbio, Cat#CA1120) was carried out. The cells were washed with PBS 3 times and then added with 500 μL staining buffer, 5 μL PI, and 5 μL Hoechst. The system was incubated at 4 °C for 30 min and then washed with PBS. The images were captured with a fluorescence microscope (OLYMPUS DP80). All images were analyzed using ImageJ to calculate the PI positive cells and Hoechst positive cells.

### Generation of *TubAR* knockdown mice

C57/B6J mice were purchased from GemPharmatech (Nanjing, China) and housed in a temperature-controlled room under a 12 h light/12 h dark cycle, with free access to food and water.

Sh*TubAR-1* and sh*TubAR-2* were designed and independently packaged into AAV by OBiO (Shanghai, China). 2-month-old mice were anesthetized by intraperitoneal injection of 0.5% pentobarbital sodium solution (0.01 mL/g). The anesthetized mice were fixed on the stereotactic instrument. The glass pipette connected with 10 μL syringe was placed in bilateral hemispheres of cerebellum (A/P: –7.1 mm, M/L: +/–1.0 mm, D/V: –3.0 mm) or M2 cortex (A/P: +1.3 mm, M/L: +/–0.7 mm, D/V: –0.8 mm). 1 μL control AAV, sh*TubAR-1* or sh*TubAR-2* virus (concentration no less than 1 × 10^8^ vg/mL) was injected into each side of the mouse cerebellum or M2 cortex with microinjection pump at a speed of 50 nL/min. The behavioral experiments were carried out 1 month after the injection. The MRI experiments were carried out 2 months after the injection.

All animal manipulations were conducted in strict accordance with the guidelines and regulations set forth by the USTC Animal Resources Center and Animal Care and Use Committee (Permission Number: USTCACUC1801023). The packaged shRNA sequences used are as follows:

shCtrl^AAV^: TTCTCCGAACGTGTCACGT; sh*TubAR*^AAV^*-1*: GCAGTTGGGATGAAAGACT; sh*TubAR*^AAV^*-2*: GCTGGACACTCAGCTCAAT.

### Rotarod test and open-field test

A total of 6 trials for the rotarod test were carried out using the rotarod training system (Xinruan XR1514) following the procedure as described before^62^. Open-field test was carried out following the procedure as described before^64^.

### MRI

The sh*TubAR*^AAV^-1 mice and shCtrl^AAV^ mice at 4-month-old (2 months post injection) were used. All MRI experiments were performed with a 7.0 Tesla 20-cm horizontal bore MR spectrometer (BRUKER BioSpec 70/20USR). 20-cm (diameter) birdcage coil and 20-mm (diameter) surface coil were used to transmit or receive MRI signals. The animals were placed in a prone position on a specially designed cradle and inserted into the magnet, fixed with two ear bars and a tooth bar. Mice were anaesthetized with isoflurane (4% induction, 1.0%–1.2% maintenance in air/O2 7:3) for the duration of the scan. T2-weighted MR images were acquired by sagittal using a rapid acquisition with relaxation enhancement (RARE) sequence with a repetition time (TR) of 3000 ms, RARE factor = 4; effective echo time (TE) = 45 ms, field of view (FOV) = 20 × 20 mm^2^, matrix size = 256 × 256, slice thickness = 0.6 mm (15 slices, gap = 0), and bandwidth (BW) = 50 kHz, number of average = 4.

### Immunofluorescence

Brain sections were permeabilized by PBS containing 0.3% Triton X-100 for 5 min followed by 40 min of blocking (3% BSA, 3% concentrated goat serum in PBS), and conducted to the IF procedure as described before^62^. The primary antibodies used were chicken anti-NFL (Abcam, Cat#ab72997, RRID: AB_1267598, 1:300), rabbit anti-CALBINDIN (Abcam, Cat#ab108404, RRID: AB_10861236, 1:100), mouse anti-CNP (Abcam, Cat#ab6319, RRID: AB_2082593, 1:300), rabbit anti-NeuN (Abcam, Cat#ab177487, RRID: AB_2532109, 1:300), or rabbit anti-vGLUT1 (Abcam, Cat#ab227805, RRID: AB_2868428, 1:100). The secondary antibodies used were Alexa Flour 568 conjugated anti-Rabbit (Abcam, Cat#ab175471, 1:500), Alexa Flour 555 conjugated anti-Chicken secondary antibody (Invitrogen, Cat#A32932, AB_2762844, 1:2000) or Alexa Flour 647 conjugated anti-Mouse secondary antibody (Invitrogen, Cat#A32728, RRID: AB_2633277, 1:2000). Images of cerebellum area were captured on an OLYMPUS FV1200MPE or LEICA STELLARIS confocal fluorescence microscope.

Quantification was performed as previously described^62^. Briefly, 4 square images of 1 × 1 mm were taken from each sample. All images were analyzed using ImageJ to calculate the fluorescence intensity. All experiments were performed 3 times independently. All the comparable treatments were simultaneously performed and analyzed on the same day.

### RNA-FISH

Cy5-labeled *TubAR*, and Cy3-labeled *U6* (marker for nuclear RNA) and *18* *S* (marker for cytoplasmic RNA) probes were designed and synthesized by GenePharma. The RNA-FISH assay was conducted according to the manufactures’ instructions (for cultured cells, GenePharma, Cat#F11201; for tissue sections, GenePharma, Cat#F31201) and performed as described previously^62^. The probes used are as follows:

*TubAR* FISH probes:

5′ Cy5-TGATTGAAATAAGGAGTCGTGTAGC-3′;

5′ Cy5-CTCGTGTGTAATGATTGACTGACTGAT-3′;

5′ Cy5-CACGATGAGAACAAGGGCTGAA-3′;

*U6* FISH probe:

5′ Cy3-CTGCCTTCCTTGGATGTGGTAGCCGTTTC-3′;

*18* *S* FISH probe:

5′ Cy3-TTTGCGTGTCATCCTTGCG-3′;

### Immuno-FISH

For tubulin extraction, cells were washed with PBS followed by PHEM buffer (60 mM PIPES, 25 mM HEPES, 10 mM EGTA, 2 mM Mg_2_Cl) then soluble tubulin heterodimers were extracted using 0.1% Triton X-100 with 10 μM taxol and 0.1% DMSO in PHEM buffer. Extracted cells were fixed with 2% PFA and 0.05% glutaraldehyde in PBS for 10 min, washed with PBS and then reduced in ethanol for 7 min at room temperature. Cells were then washed with PBS and blocked in 3% BSA and 0.2% Triton X-100 in PBS for 20 min at room temperature. Then, the cells were incubated with Mouse anti- alpha Tubulin (DM1A; Cat#ab7291, RRID: AB_2241126, 1:300) 1 h at room temperature. The cells were washed in PBS and incubated with Alexa Flour 488 labeled anti-Mouse secondary antibody (Invitrogen, Cat#A32723, RRID: AB_2633275, 1:2000) for 1 h at room temperature. RNA-FISH was then conducted as described before. After three times PBS wash, cell nuclei were stained by Hoechst 33342 (Invitrogen, Cat#H3570) for 10 min. The cells were sealed by 75% glycerol/PBS. Images were captured on a LEICA STELLARIS confocal fluorescence microscope.

### In vitro transcription

PcDNA3.1(+)-*TubAR* and *TubAR AS* plasmids were linearized by Sal I enzyme as the in vitro transcription template. The RNA products were transcribed by a T7 RNA polymerase kit in vitro (Invitrogen, Cat#18033100), treated with RNase-free TURBO DNase (Invitrogen, Cat#AM2239). *TubAR* and *TubAR AS* were purified (Magen, Cat#R2144-03) and further used in RNA pull-down and microtubule co-sedimentation assay.

### RNA pull-down

In vitro transcribed *TubAR* was labeled with the Pierce RNA 3′ End Desthiobiotinylation Kit (Thermo Scientific, Cat#20163). The resulted 3′ desthiobiotinlabelled *TubAR* or a negative control RNA provided by the kit was then incubated with Streptavidin-coated Dynabeads M-280 (Invitrogen, Cat#11205D) for 30 min at room temperature. Parallelly, cerebellum lysates were obtained and resolved in Protein-RNA Binding Buffer (0.2 M Tris-HCl (pH 7.5), 0.5 M NaCl, 20 mM MgCl_2_, 1% Tween-20) in the presence of 50% glycerol. 10% input was saved and the rest was subjected for the following steps. The cerebellum lysates and prepared 3′ desthiobiotin-labeled RNA conjugated-beads were further incubated at 4 °C for 2 h with rotation. After wash for six times, the *TubAR*-interacting proteins were collected and separated by SDS-PAGE. The specific bands of *TubAR* to control RNA were cut and subsequentially analyzed by gas chromatography-mass spectrometry (GC/MS, Agilent 5975MSD). The interested targets were further validated by western blotting assay.

### Plus-end microtubule binding assay

HeLa cells were seeded at a density of 1 × 10^6^ cells per well on glass bottom dish and co-transfected with 2.5 µg of EB3-EGFP and *TubAR* or *TubAR AS* respectively. 48 h after transfection, the cells on the plate were placed on a ZEISS LSM 980 confocal live cell station. Images were captured every 2 s for 1 min in total as described before with some modification^46^. Time lapse was analyzed by ImageJ software to calculate EB3 comet velocity to indicate the speed of microtubule extension. A video was also integrated by ImageJ software. Assays were performed six times with experimental duplicates.

### Microtubule co-sedimentation assay

Microtubule co-sedimentation assay was performed as described previously^65^. Briefly, the unlabeled tubulin mix (Cytoskeleton, T240-B) was diluted in BRB80 buffer (80 mM PIPES, 1 mM MgCl_2_, 1 mM EGTA, pH 6.8 with KOH). Next, the purified *TubAR* (final concentration: 0.1 nM) or *TubAR AS* (final concentration: 0.1 nM) was added to polycarbonate centrifuge tubes (BECKMAN COUNTER, 343778) in the presence of the diluted tubulin mix, GMPCPP (final concentration: 1 mM, Jena Bioscience, NU4055), and paclitaxel (final concentration: 1.5 μM). A reaction without tubulin mix was included as negative control for *TubAR* and *TubAR AS*. All the reactions were further incubated at 37 °C for 30 min. The polymerized and unpolymerized tubulins were separated by ultracentrifugation at 90,000 rpm for 10 min. The supernatant (unpolymerized tubulins) or the pellet (polymerized tubulins) was collected following with Coomassie blue staining or RT-qPCR.

### RIP assay

Cells were harvested by trypsinization and resuspended in PBS and cross-linked with 1% formaldehyde for 10 min at room temperature. Cross-linking was stopped by adding glycine to a final concentration of 0.25 M at room temperature for 5 min. Cell pellets were collected by centrifugation at 1000 rpm for 5 min and lysed by NP-40 Lysis Buffer (Beyotime, Cat#P0013F) containing PMSF, Proteinase Inhibitor Cocktail (Roche, Cat#4693132001) and RNase Inhibitor (Vazyme, Cat#R301-03-AA). After sonication for 6 min (6 s on/6 s off each time, SCIENTZ JY92IIN), the soluble fraction was collected after centrifugation at 12,000 rpm for 10 min at 4 °C. The soluble fraction was pre-cleared with 50 μL Protein A/G Agarose beads (Santa Cruz, Cat#sc-2001) at 4 °C for 1 h on rotator. 10% input was saved and the rest was subjected for the following steps. While waiting, desired antibodies were conjugated with Protein A/G Agarose beads. The pre-cleared soluble fraction was then added to the eppendorfs with the conjugated beads at 4 °C for 6 h on rotator. Next, the beads were washed six times using NP-40 Lysis Buffer. Finally, appropriate amount of TRIzol Reagent was added to extract the protein-RNA complex for qPCR detection. The antibodies used for RIP assay are anti-FLAG (Abcam, Cat#ab125243, RRID: AB_11001232), anti-GFP (Proteintech, Cat#66002-1-Ig), anti-TUBA1A (Abcam, Cat#ab200216), Rabbit IgG (Abcam, Cat#ab172730), and Mouse IgG (Santa Cruz, Cat#sc-2025).

In sequential RIP Assay, the beads-1st round antibody–protein-RNA complex obtained by immunoprecipitation described under RIP assay was eluted in 50 μL 10 mM DTT at 37 °C for 30 min. The supernatant was collected by centrifugation and further diluted 10 times with NP-40 Lysis Buffer containing PMSF, Proteinase Inhibitor Cocktail and RNase Inhibitor. 10% input was saved. The rest was subjected to repeating the RIP procedure in the presence of 2^nd^ round antibody, followed by RNA extraction and detection.

### Co-IP

Cells were harvested and lysed at 4 °C for 30 min in NP-40 Lysis Buffer containing PMSF, Proteinase Inhibitor Cocktail, and RNase Inhibitor. The supernatants were collected by centrifugation at 12,000 rpm for 10 min at 4 °C and pre-cleared with 50 μL Protein A/G Agarose beads at 4 °C for 1 h on rotator. 10% lysis was saved as input and the rest was subjected for the following steps. While waiting, anti-GFP and the control Mouse IgG were conjugated to Protein A/G Agarose beads. The pre-cleared supernatants were added to the antibody conjugated beads and rotated at 4 °C overnight. The beads were washed six times using IP Wash Buffer (10 mM Tris pH 7.4, 1 mM EDTA, 150 mM NaCl, 1% Triton X-100) containing PMSF and Proteinase Inhibitor Cocktail. Next, the protein complex was dissolved in the protein loading buffer (1 M Tris-HCl pH 6.8, 25% glycerol, 10% SDS, 0.025% BPB, 2.5% β-ME) and subjected to SDS-PAGE for western blotting assay. RNase A treatment was conducted to test the effect of RNA in TUBB4A–TUBA1A interaction in Fig. 4i–k.

### Data analysis

All data are presented as means ± SEM. All statistical analyses were performed using the GraphPad Prism Software. For comparisons, tests for rotarod were carried out using the mixed-effects model with Geisser–Greenhouse correction; other data were analyzed using Student’s *t*-test with Welch’s correction. *P* < 0.05 was considered significant.

### Supplementary information

Supplementary Information

Supplementary Video S1-S3

## Data Availability

The data that support the findings of this study are available upon reasonable request.
