# Supplementary material for: LncRNA TubAR complexes with TUBB4A and TUBA1A to promote microtubule assembly and maintain myelination
Source: Cell Discov. 2024 May 21;10:54. doi: 10.1038/s41421-024-00667-y (PMC11106304; doi:10.1038/s41421-024-00667-y)
Supplement: Supplementary file 1 — Supplementary Information [file 41421_2024_667_MOESM1_ESM.pdf]

LIANG_Supplementary Table S1

List of tubulins from RNA Pull-down/MS (cerebellum)

Name	Peptides	PSM	Coverage	Description
TUBA1A	12	16	37	tubulin alpha-1a chain
TUBA4A	10	12	29	tubulin alpha-4a chain
TUBB2A	13	26	37	tubulin beta-2a chain
TUBB2B	13	25	37	tubulin beta-2b chain
TUBB4A	12	25	34	tubulin beta-4a chain
TUBB4B	13	27	36	tubulin beta-4b chain
TUBB5	13	27	36	tubulin beta-5 chain

PSM: Peptide Spectrum Match

LIANG_Supplementary Table S2

List of tubulins from RNA Pull-down/MS (HeLa cells)

Name	Peptides	PSM	Coverage	Description
TUBA1A	23	88	60	tubulin alpha-1a chain
TUBA1C	23	90	60	tubulin alpha-1c chain
TUBA4A	22	76	58	tubulin alpha-4a chain
TUBB2A	18	84	44	tubulin beta-2a chain
TUBB2B	18	88	44	tubulin beta-2b chain
TUBB4A	21	115	57	tubulin beta-4a chain
TUBB4B	21	106	56	tubulin beta-4b chain
TUBB6	13	40	37	tubulin beta-6 chain

PSM: Peptide Spectrum Match

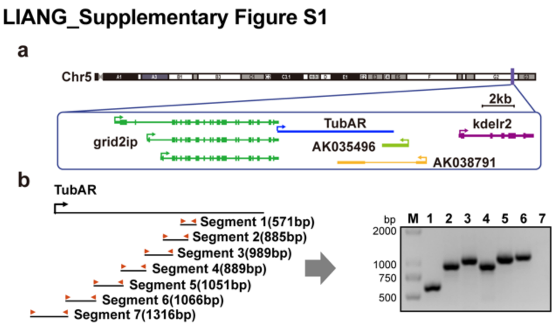

Supplementary Figure S1. Segment RT-PCR for *TubAR*, related to Figure 1.

(a) Chromosome location of *TubAR* and *TubAR* neighboring genes. Modified from UCSC mm10 database.

(b) Schematic Map (left) and Electrophonic Gel Image (right) of Segment RT-PCR for *TubAR*.

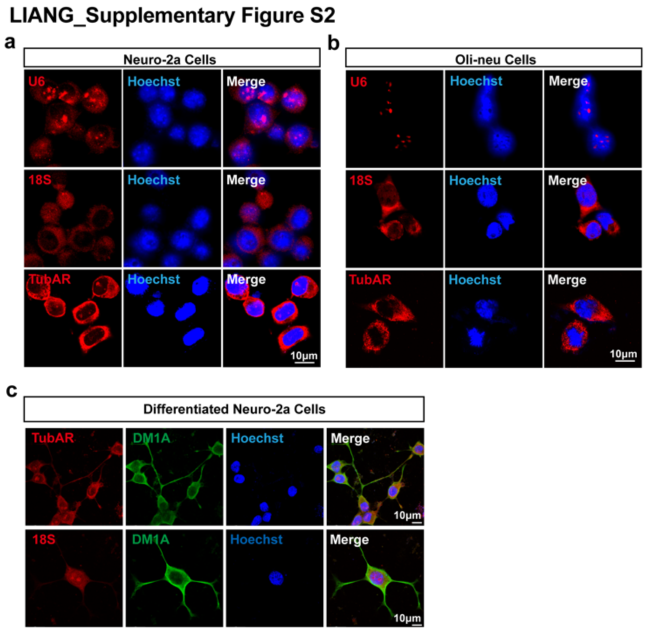

Supplementary Figure S2. FISH analyses of *TubAR* show cytoplastic expression in proliferating Neuro-2a cells (a), Oli-neu cells (b), and differentiated Neuro-2a cells (c), related to Figure 1.

U6: marker for nuclear RNA; *18S*: marker for cytoplasmic RNA. Scale bar, 10 μm.

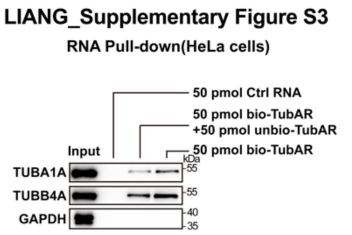

Supplementary Figure S3. Competitive RNA pull-down of *TubAR* using HeLa cell extracts, related to Figure 2.

Competitive RNA pull-down assay to validate the interactions of *TubAR* with TUBA1A and TUBB4A, using HeLa cell extracts. bio-TubAR, biotinylated *TubAR*; unbio-TubAR, unbiotinylated *TubAR*; GAPDH was used as a negative control for RNA-protein interaction.

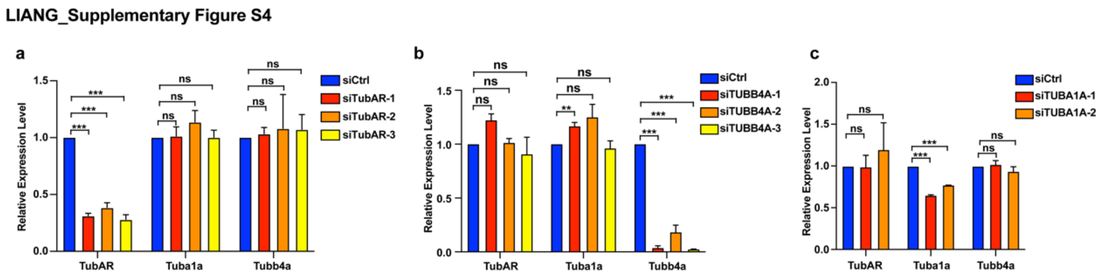

Supplementary Figure S4. Knockdown efficiency of the indicated siRNAs and their effects on the expression levels of the related factors, related to Figures 2, 3, and 6.

(a) The expression levels of *TubAR*, *Tuba1a*, and *Tubb4a* in Neuro-2a cells expressing the indicated siCtrl or si*TubAR*s (n=3). All data were normalized to *Gapdh*.

(b) The expression levels of *TubAR*, *Tuba1a* and *Tubb4a* in Neuro-2a cells expressing the indicated siCtrl or siTUBB4As (n=3). All data were normalized to *Gapdh*.

(c) The expression levels of *TubAR*, *Tuba1a*, and *Tubb4a* in Neuro-2a cells expressing the indicated siCtrl or siTUBA1As (n=3). All data were normalized to *Gapdh*.

Data are represented as means ± SEM. Statistical significance was determined using unpaired t-test with Welch's correction; ns, P > 0.05; **P < 0.01; ***P < 0.001.

LIANG_Supplementary Figure S5

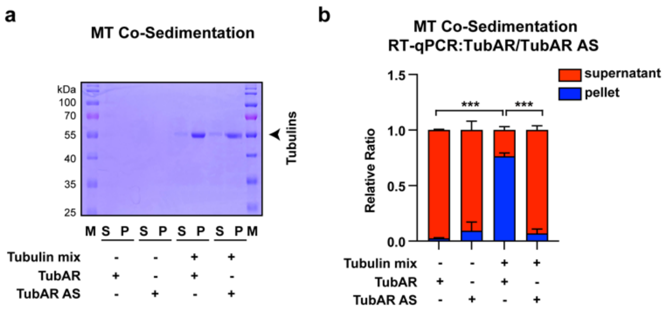

Supplementary Figure S5. *TubAR* co-precipitates with microtubules by co-sedimentation assay, related to Figure 3.

(a) The distributions of tubulins in the pellet (P) or supernatant (S) following microtubule co-sedimentation detected by Coomassie brilliant blue staining.

(b) Quantification of the indicated RNAs in the pellet or supernatant following microtubule co-sedimentation detected by RT-qPCR (n=3).

LIANG_Supplementary Figure S6

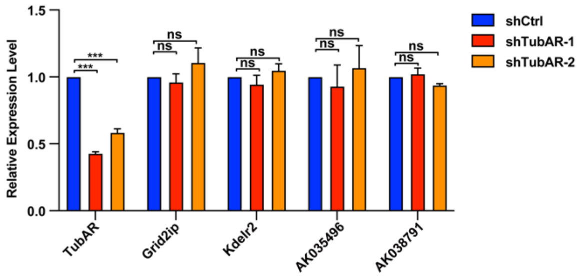

Supplementary Figure S6. Effects of *TubAR* knockdown on itself and expression level of *TubAR*-neighboring genes, related to Figure 7.

The expression levels of *TubAR*, and its neighboring genes (*Grid2ip*, *Kdelr2*, *AK035496* and *AK038791*) under shCtrl and the indicated sh*TubAR*s transfected cells by qPCR (n=3).

All data were normalized to the *Gapdh*. Data are represented as means ± SEM. Statistical significance was determined using unpaired t-test with Welch's correction; ns, P > 0.05; ***P < 0.001.

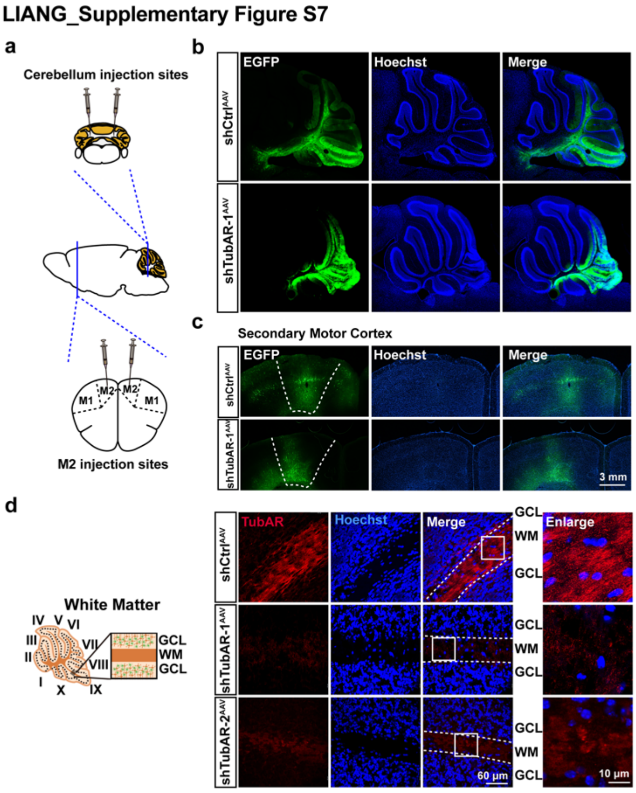

Supplementary Figure S7. Mice injection strategy and virus spreading regions, related to Figure 7.

(a) Illustration of virus injection sites of cerebellum (top) or secondary motor cortex (M2; bottom).

(b) Virus spreading regions from the cerebellum-injected mice indicated by EGFP. Slice thickness, 40 μm.

(c) Virus spreading regions from the secondary motor cortex-injected mice indicated by EGFP. Slice thickness, 40 μm; Scale bar, 3 mm.

(d) Knockdown efficiency of sh*TubAR*^{AAV}s in white matter of cerebellum-injected mice assessed by *TubAR* FISH. Scale bar, 60 μm; Slice thickness, 6 μm.

AAV, adeno-associated virus.

LIANG_Supplementary Figure S8

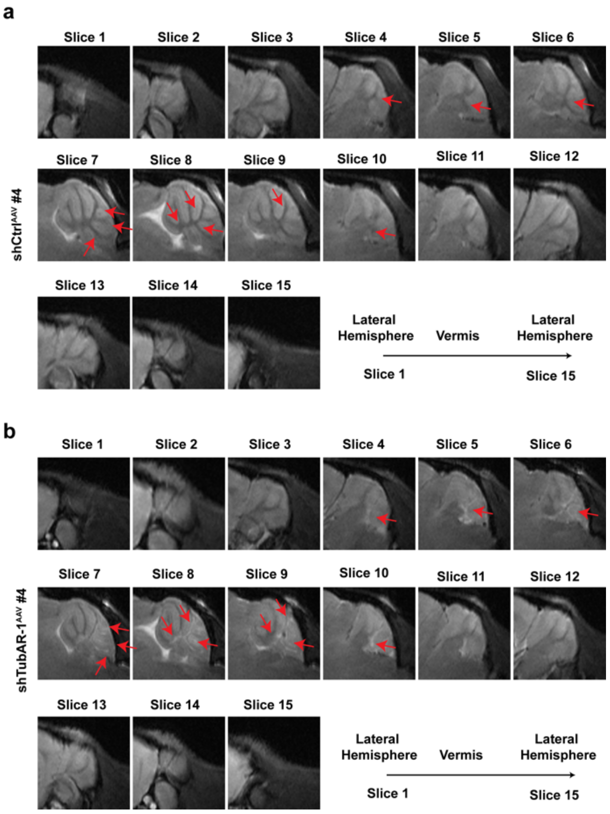

Supplementary Figure S8. Cerebellum-specific knockdown of *TubAR* results in demyelination, related to Figure 7.

(a, b) MRI images of all 15 slices for representative shCtrl^{AAV}#4 and sh*TubAR*-1^{AAV}#4. Slices 7-9 were the center of cerebellar vermis. Arrows on Slices 4-10 indicate areas with altered MRI signals between sh*TubAR*-1^{AAV} and shCtrl^{AAV} mice.

AAV, adeno-associated virus.

Supplementary Video S1: The presence of *TubAR* causes a significantly slower kinetics of microtubule assembly, related to Figure 3.

Supplementary Video S2: Knockdown of TUBA1A causes a significantly faster kinetics of microtubule assembly, related to Figure 3.

Supplementary Video S3: Knockdown of TUBB4A causes a significantly faster kinetics of microtubule assembly, related to Figure 3.
