# Supplementary material for: LncRNA TubAR complexes with TUBB4A and TUBA1A to promote microtubule assembly and maintain myelination
Source: Cell Discov. 2024 May 21;10:54. doi: 10.1038/s41421-024-00667-y (PMC11106304; doi:10.1038/s41421-024-00667-y)
Supplement: Supplementary file 2 — Supplementary Video S1-S3 [file 41421_2024_667_MOESM2_ESM.pptx]

## Slide 1
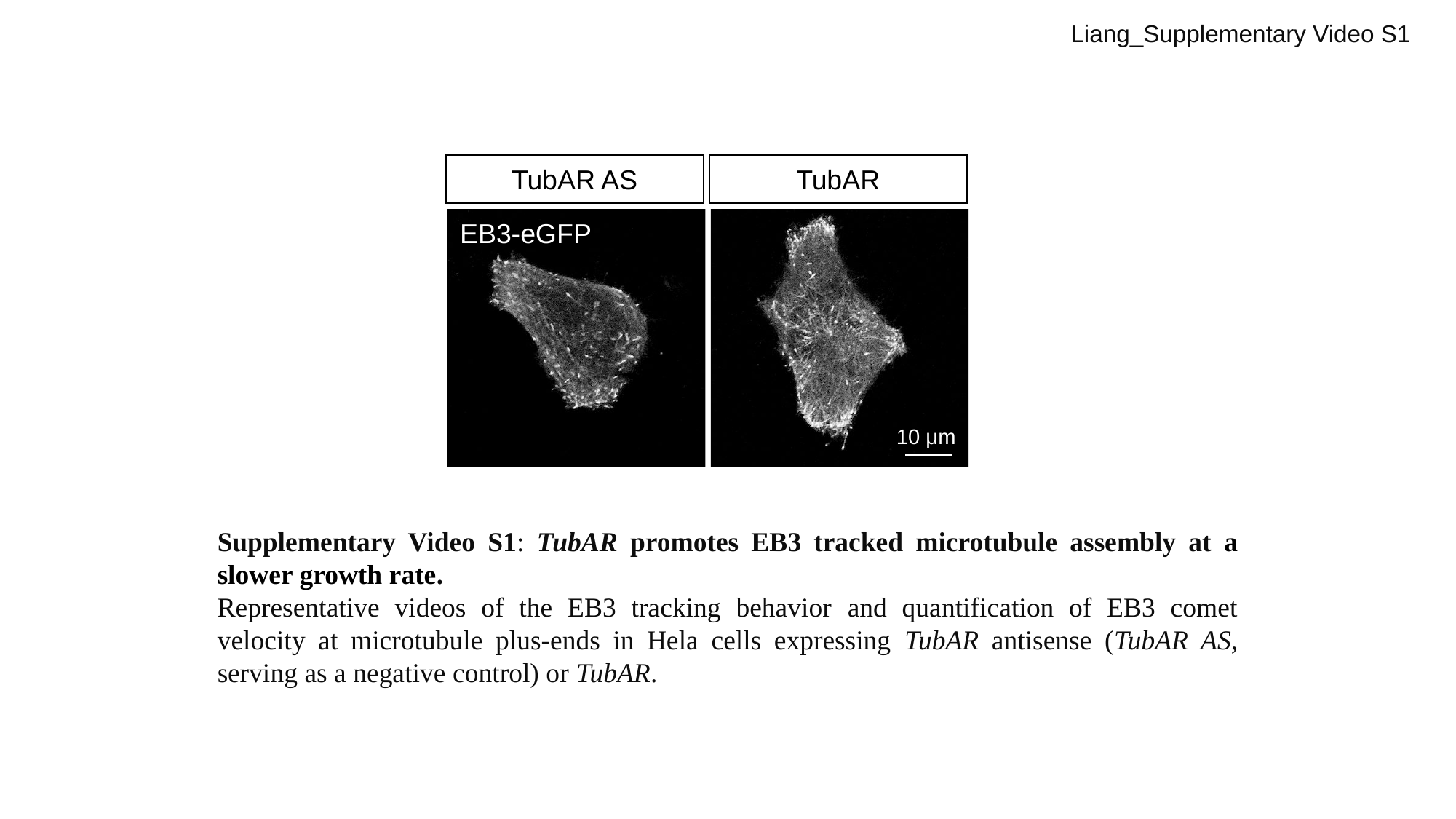

Liang_Supplementary Video S1
TubAR AS
TubAR
EB3-eGFP
10 μm
Supplementary Video S1: TubAR promotes EB3 tracked microtubule assembly at a slower growth rate.
Representative videos of the EB3 tracking behavior and quantification of EB3 comet velocity at microtubule plus-ends in Hela cells expressing TubAR antisense (TubAR AS, serving as a negative control) or TubAR.

## Slide 2
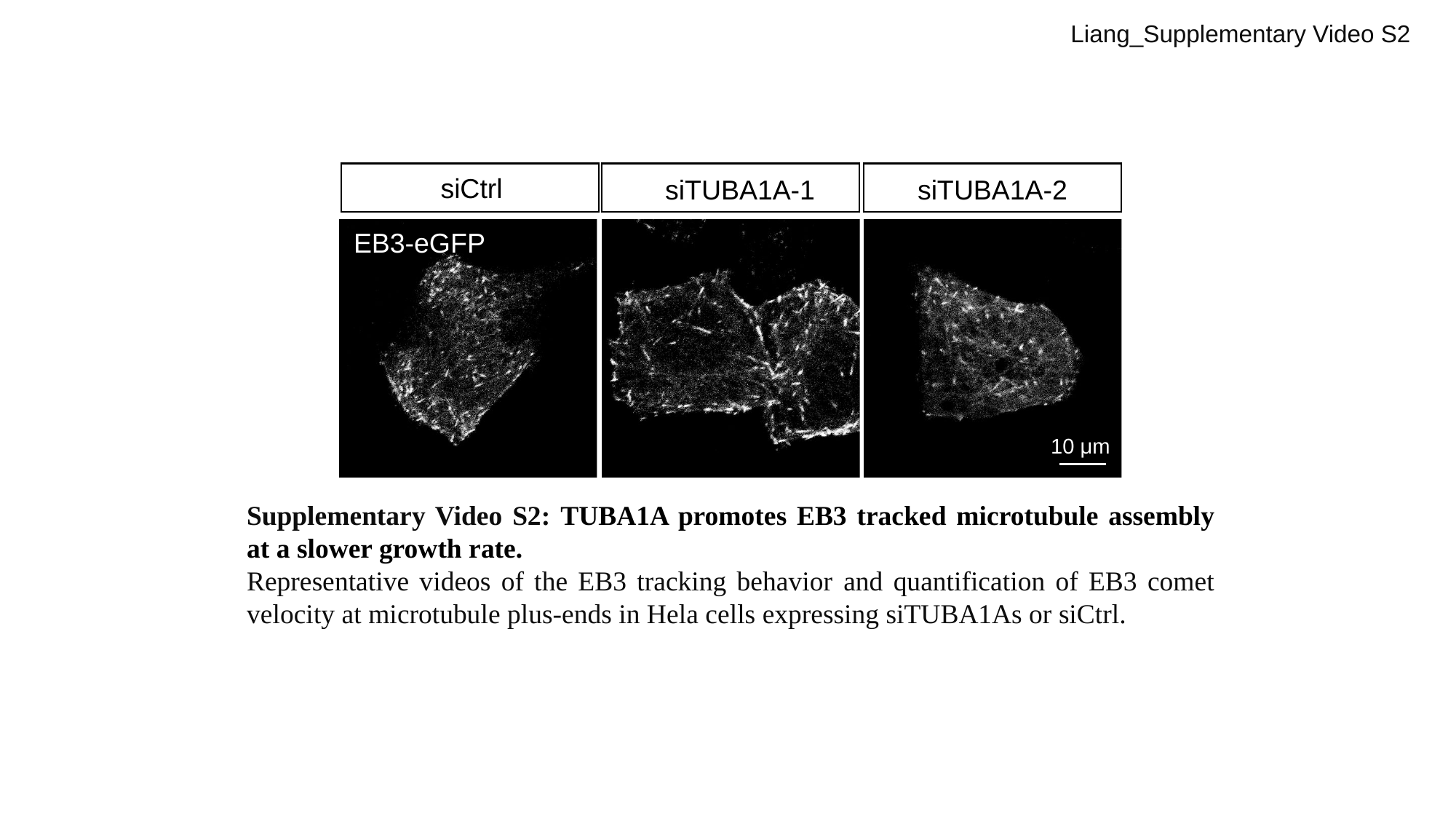

Liang_Supplementary Video S2
siCtrl
siTUBA1A-1
siTUBA1A-2
EB3-eGFP
10 μm
Supplementary Video S2: TUBA1A promotes EB3 tracked microtubule assembly at a slower growth rate.
Representative videos of the EB3 tracking behavior and quantification of EB3 comet velocity at microtubule plus-ends in Hela cells expressing siTUBA1As or siCtrl.

## Slide 3
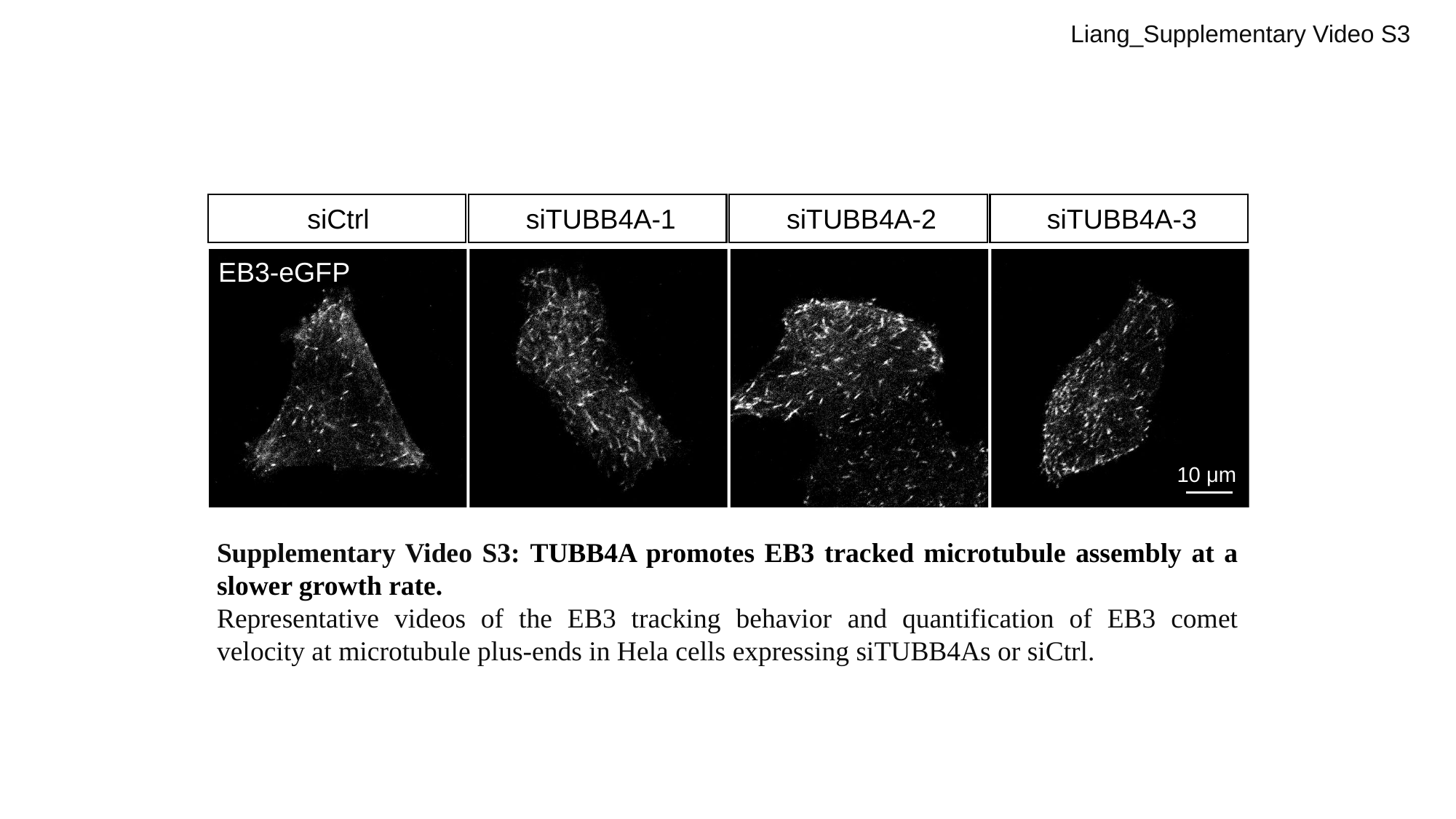

Liang_Supplementary Video S3
siCtrl
siTUBB4A-1
siTUBB4A-2
siTUBB4A-3
EB3-eGFP
10 μm
Supplementary Video S3: TUBB4A promotes EB3 tracked microtubule assembly at a slower growth rate.
Representative videos of the EB3 tracking behavior and quantification of EB3 comet velocity at microtubule plus-ends in Hela cells expressing siTUBB4As or siCtrl.
